# Energy spectrum-based correction of washout rates in dual-isotope ^123^I-BMIPP/^201^Tl imaging

**DOI:** 10.1007/s12149-026-02235-8

**Published:** 2026-05-30

**Authors:** Kenichi Nakajima, Shotoku Makida, Masato Okamura, Masa-aki Kawashiri, Takayuki Shibutani

**Affiliations:** 1 Department of Nuclear Medicine, Kanazawa University, 13-1 Takara-Machi, Kanazawa, 920-8640 Japan; 2 Department of Nuclear Medicine, Kaga Medical Center, Kaga, Japan; 3 Department of Cardiology, Kaga Medical Center, Kaga, Japan; 4 Present Address: Department of Cardiology, Public Central Hospital of Matto Ishikawa, Hakusan, Japan; 5 Department of Radiation Technology, Kaga Medical Center, Kanazawa, Japan; 6 Department of Quantum Medical Technology, Institute of Medical, Pharmaceutical and Health Sciences, Kanazawa University, Kanazawa, Ishikawa Japan

**Keywords:** nuclear cardiology, fatty acid imaging, ischemic heart disease, cardiomyopathy, ^123^I-labeled radiopharmaceuticals

## Abstract

**Background:**

Myocardial metabolism and perfusion in patients with ischemic heart disease or cardiomyopathy can be simultaneously assessed using ^123^I-β-methyl-iodophenyl-pentadecanoic acid (BMIPP) and ^201^Thallium (Tl) dual-isotope images. However, cross-contaminated counts between these tracers complicate quantitative analyses, particularly of ^123^I-BMIPP washout rates (WRs). We developed a simplified method to determine WRs corrected for energy spectral crosstalk from dual-isotope images.

**Methods:**

Our correction method was based on fixed energy-spectrum spill-in ratios (iRatio for ^123^I and tRatio for ^201^Tl) that were empirically determined from phantom and patient datasets. These ratios were applied to separate ^123^I and ^201^Tl counts in 38 studies of 36 patients with ischemic heart disease using dual-energy planar and single-photon emission computed tomography (SPECT) images. We calculated WRs before and after correction using early and late anterior planar images, as well as mean tomographic counts derived from polar maps. Thresholds of 5% (planar) and 10% (SPECT) were used to assess classification agreement between corrected and uncorrected WRs.

**Results:**

The range of iRatios was 0.57–0.73 and that of tRatios was 0.10–0.12. Higher iRatio values were associated with greater body-weight patients, whereas tRatios were relatively stable regardless of body weight. The corrected ^123^I-BMIPP WR depends primarily on tRatio and, by formulation, is not directly affected by iRatio. Using a fixed tRatio of 0.12, the correction method revealed strong correlations between corrected and uncorrected WRs for planar imaging (R² = 0.969, *p* < 0.0001) and SPECT (R² = 0.964, *p* < 0.0001). Corrected ^123^I-BMIPP WRs were comparable to uncorrected values for both planar imaging (mean difference, − 1.4%) and SPECT (mean difference, 0.8%). Among the 38 studies, classification of tentative normal versus abnormal WRs remained concordant in 35 (92%) planar studies and 36 (95%) SPECT studies.

**Conclusion:**

Our simplified energy spectrum–based method demonstrated that correction of ^123^I-BMIPP WRs is feasible under dual-isotope conditions. Although uncorrected WRs may provide approximate estimates, correction is important for reliable quantitative use of dual-isotope datasets, where spectral cross-talk limits accuracy. The proposed model-based approach improves the robustness and interpretability of WR assessment and enables practical, standardized quantitation.

**Supplementary Information:**

The online version contains supplementary material available at https://doi.org/10.1007/s12149-026-02235-8.

## Introduction

Dual-isotope SPECT with ^123^I-β-methyl-iodophenyl pentadecanoic acid (^123^I-BMIPP) and ^201^Tl-chloride (^201^Tl) [1–3] has been used to simultaneously evaluate myocardial fatty acid metabolism and perfusion in patients with coronary artery disease and heart failure. Previous visual assessments of matched and mismatched uptake patterns between these tracers have provided critical insights into myocardial viability, especially during the early phase after acute ischemic events. Early mismatches between preserved perfusion (^201^Tl) and reduced fatty acid uptake (^123^I-BMIPP) are associated with a viable but ischemic myocardium and may predict functional recovery [4, 5]. Despite the clinical value of dual-tracer imaging for viability assessment, most applications remain qualitative, with limited consideration of quantitative parameters such as ^123^I-BMIPP washout rates (WRs).

Accurate quantitation of myocardial dual-isotope radiotracer clearance, typically expressed as WRs between early and late images, is important for diagnostic assessment and for understanding myocardial pathophysiology [6, 7]. However, the physical characteristics of these radionuclides complicate quantitative analysis. Whereas ^123^I emits a principal gamma photon at 159 keV, ^201^Tl emits characteristic mercury X-rays around 70 keV and additional gamma photons at 167 keV. The 167 keV component of ^201^Tl partially overlaps the ^123^I 159 keV photopeak, particularly when energy windows such as 15%–30% are relatively wide. Despite this crosstalk, practical and standardized correction strategies applicable to routine clinical imaging remain limited.

Washout rates of ^123^I-BMIPP images are key quantitative parameters for diagnosing triglyceride deposit cardiomyovasculopathy (TGCV), for which a WR threshold of 10% is a common diagnostic criterion [8, 9]. Although WRs can be calculated in various ways [10–12], the specific impact of dual-isotope acquisition on WR accuracy has not been systematically evaluated. This limitation is particularly relevant in routine dual-tracer imaging, where spectral overlap between ^123^I and ^201^Tl can bias measured WRs.

The primary aims of this study were to evaluate the feasibility of determining ^123^I-BMIPP WRs under dual-isotope acquisition conditions, to develop a simple energy spectrum–based method for separating tracer signals, and to quantify the bias introduced by dual-isotope imaging. We further propose a practical correction approach to estimate WRs for ^123^I-BMIPP and to explore its applicability to ^201^Tl using planar and single-photon emission computed tomography (SPECT) images.

### Methods

#### Data acquisition

Syringes containing ^123^I-BMIPP and ^201^Tl solutions (111 MBq each) were placed in a cylindrical acrylic phantom (diameter, 13 cm) designed to simulate the human neck and an upper insert to represent the thyroid gland. Energy spectra were acquired using a dual-head gamma camera (NM830, GE Healthcare, Tokyo, Japan) equipped with extended low-energy general-purpose (ELEGP) collimators. Energy spectra of ^123^I, ^201^Tl, and dual-tracer (^123^I + ^201^Tl) were acquired for 3 min. These datasets were used to characterize energy-spectrum crosstalk and to establish spill-in ratios for correcting dual-isotope images.

### Patients

We acquired ^201^Tl and ^123^I-BMIPP images from 36 consecutive patients (38 studies; mean age, 69 ± 10 y; male, *n* = 22) with ischemic heart disease. Baseline diagnoses included acute myocardial infarction after percutaneous coronary intervention (*n* = 12; 14 studies), old myocardial infarction (*n* = 7), vasospastic angina (*n* = 2), myocardial infarction with non-obstructive coronary arteries (*n* = 2), cardiomyopathy (*n* = 2), heart failure (*n* = 1), and atypical or typical chest discomfort (*n* = 10). All data were anonymized before analysis, and identifiable patient information was neither accessed nor stored. The Ethics Committee at Kaga Medical Center approved this technical evaluation of dual-tracer correction using anonymized clinical data (Approval No.: study 2025 [R7]-7). The need for patient consent was waived due to the retrospective study design.

### Planar and SPECT imaging

Planar and SPECT images were acquired from all patients at 20 min (early) and at 3 h (late) after intravenous injections of ^201^Tl and ^123^I-BMIPP (111 MBq each). Anterior planar images were acquired using a 128 × 128 matrix for 5 min per frame under a dual-energy acquisition protocol. Energy windows were centered at 71 keV (30%) for ^201^Tl and 159 keV (20%) for ^123^I. Subsequent SPECT images were acquired over a 180° arc. Projection data were acquired using a 64 × 64 matrix, and tomographic images were reconstructed using filtered back projection (FBP) with a Butterworth pre-filter (cutoff 0.45 cycles/cm, order 10) and ordered-subset expectation maximization (OSEM; 2 iterations, 10 subsets, Butterworth post-filter) algorithms. Energy spectra were extracted from quality control (QC) XML files of a subset of seven patients, at early and late time points, to confirm spectral consistency in vivo.

### Creation and calculation of separate and combined energy spectra

Separate ^123^I and ^201^Tl energy spectra were acquired to simulate combined dual-radionuclide conditions. The relative intensities were adjusted to match approximate peak counts at 71 keV (^201^Tl) and 159 keV (^123^I) to mimic clinical acquisition conditions (Fig. 2A). The main energy windows were defined as ± 10% (20% total width) and ± 15% (30% total width) for ^123^I and ^201^Tl, centered at 159 and 71 keV, respectively, consistent with standard clinical protocols.

Counts extracted from the spectra are presented as C (radionuclide, window), where I and T denote ^123^I and ^201^Tl, and wI and wT denote the ^123^I and ^201^Tl windows, respectively. The following variables were defined as:

*C*_*I, wI*_, *C*_*I, wT*_ represent ^123^I counts in the ^123^I and ^201^Tl windows, respectively; *C*_*T, wI*_, *C*_*T, wT*_ represent ^201^Tl counts in ^123^I and ^201^Tl windows, respectively.

**Fig. 1:**
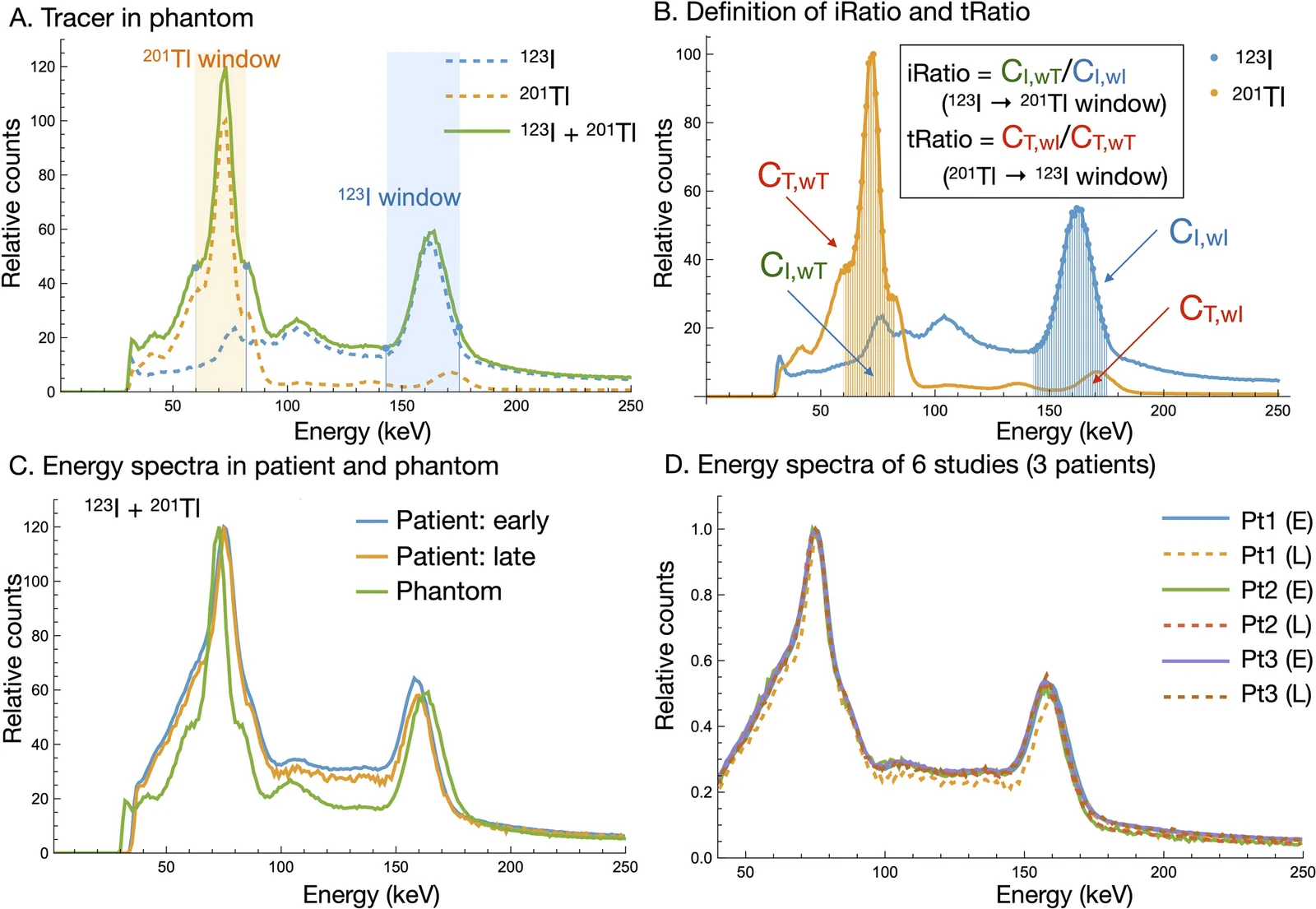
Energy spectra of ^123^I, ^201^Tl, and dual-isotope ^123^I/^201^Tl acquisition in phantom and clinical studies. (**A**) Energy spectra acquired from a phantom under single- and dual-isotope conditions (^123^I, ^201^Tl, and both ^123^I + ^201^Tl). (**B**) Definition of spill-in iRatio and tRatio based on counts in ^201^Tl and ^123^I energy windows. (**C**) Comparison of dual-isotope spectra between the phantom and a representative patient during early and late imaging phases. (**D**) Dual-isotope energy spectra from six studies of three patients, each acquired at early (E) and late (L) phases. Shaded areas in panels A and B indicate acquisition windows. ^123^I-BMIPP, ^123^I-β-methyl-iodophenyl-pentadecanoic acid; i, iodine; Pt, patient; t, thallium

We then used these definitions to calculate spectral contamination “spill-in” ratios as: *iRatio* = *C*_*I, wT*_ */C*_*I, wI*_ and *tRatio* = *C*_*T, wI*_ */C*_*T, wT*_, representing spill-in of ^123^I into the ^201^Tl window and of ^201^Tl into the ^123^I window, respectively (Fig. 1B).

Combined energy spectra generated from phantom data were compared with patient-derived spectra obtained from QC files to confirm the consistency and applicability of the simulated dual-tracer spectra.

Energy spectra under dual-nuclide conditions were analyzed in three patients (six studies) at early and late time points. In addition, five patients underwent sequential acquisitions under single-nuclide conditions (two patients with ^123^I first and three with ^201^Tl first). Spectra were acquired again after dual-isotope administration, and the individual ^123^I and ^201^Tl components were estimated by subtracting the corresponding single-nuclide spectra from the dual-nuclide spectra.

### Separation of ^201^Tl and ^123^I counts

When counts were measured during dual-energy acquisition, each energy window represented a mixture of emissions from both radionuclides due to spectral overlap. Therefore, the measured counts in the ^201^Tl ($\:{C}_{Dual,wT}$) and the ^123^I ($\:{C}_{Dual,wI}$) windows under dual-tracer conditions were approximated as follows. A step-by-step mathematical derivation of the correction equations is provided in the Supplementary Material.

Counts in ^123^I window:

1.1 $$\:{C}_{Dual,wI}=\:{C}_{I,wI}+\:{C}_{T,wI}\approx\:{C}_{I}+\:{C}_{T}\cdot\:tRatio$$

Counts in ^201^Tl window:

1.2 $$\:{C}_{Dual,wT}=\:{C}_{T,wT}+\:{C}_{I,wT}\approx\:\:{C}_{T}+\:{C}_{I}\cdot \:iRatio$$

where $\:{C}_{I}$ is the true ^123^I count in the ^123^I window ($\:{C}_{I,wI}$) and $\:{C}_{T}$ is the true ^201^Tl count in the ^201^Tl window ($\:{C}_{T,wT}$). The spill-in ratios are defined as:

$$\:iRatio\:=\frac{{C}_{I,wT}}{{C}_{I,wI}}\:,\:tRatio\:=\frac{{C}_{T,wI}}{{C}_{T,wT}}$$

Based on these definitions, the dual-window counts can be expressed as:

2.1 $$\:{C}_{I,Dual}=\:{C}_{I}+{C}_{T}\cdot\:\:tRatio$$

2.2 $$\:{C}_{T,Dual}=\:{C}_{T}+{C}_{I}\cdot\:\:iRatio$$

Solving this linear system yields the isolated true counts

3.1 $$\:{C}_{I}=\frac{{C}_{I,Dual}-\:tRatio\:\cdot \:{C}_{T,Dual}}{1\:-\:iRatio\:\cdot \:tRatio}$$

3.2 $$\:{C}_{T}=\frac{{C}_{T,Dual}-\:iRatio\:\cdot \:{C}_{I,Dual}}{1\:-\:iRatio\:\cdot \:tRatio}$$

### Calculation of apparent and true washout rates

We calculated WRs using early (E) and late (L) variables, namely $\:{C}_{T,Dual,E}$, $\:{C}_{T,Dual,L}$ for ^201^Tl, and $\:{C}_{I,Dual,E}$ and $\:{C}_{I,Dual,L}$ for ^123^I-BMIPP, with decay correction applied to late counts. The apparent WRs (without spectral correction) for ^123^I (*IWR*_*app*_) and ^201^Tl (*TWR*_*app*_) were calculated as:

4.1 $$\:IW{R}_{app}=\frac{{C}_{I,Dual,E}-{C}_{I,Dual,L}\cdot \:DC{F}_{I}}{{C}_{I,Dual,E}}$$

4.2 $$\:TW{R}_{app}=\frac{{C}_{T,Dual,E}-{C}_{T,Dual,L}\cdot \:DC{F}_{T}}{{C}_{T,Dual,E}}$$

The decay correction factor (DCF) was defined as:

4.3 $$\:DCF=\frac{1}{{0.5}^{\frac{\varDelta\:t}{HL}}}$$

where HL is the physical half-life (13 h for ^123^I and 73 h for ^201^Tl), and Δt is the time interval between early and late scans (hours). After spill-in correction using the spectral separation model, the corrected WRs were calculated from the estimated separated counts:

5.1 $$\begin{aligned}\:\:&IW{R}_{estimated}\\&=\frac{{C}_{I,E}-{C}_{I,L}\cdot \:DC{F}_{I}}{{C}_{I,E}}\:\:\\&=\frac{\left({C}_{I,Dual,E}-{C}_{I,Dual,L}\cdot \:DC{F}_{I}\right)-tRatio\:\cdot \:\left({C}_{T,Dual,E}-{C}_{T,Dual,L}\cdot \:DC{F}_{I}\right)}{{C}_{I,Dual,E}-tRatio\:\cdot \:{C}_{T,Dual,E}}\end{aligned}$$

5.2 $$\begin{aligned}\:\:\:&TW{R}_{estimated}\\&=\frac{{C}_{T,E}-{C}_{T,L}\cdot \:DC{F}_{T}}{{C}_{T,E}}\:\\&=\frac{\left({C}_{T,Dual,E}-{C}_{T,Dual,L}\cdot \:DC{F}_{T}\right)-iRatio\:\cdot \:\left({C}_{I,Dual,E}-{C}_{I,Dual,L}\cdot \:DC{F}_{T}\right)}{{C}_{T,Dual,E}-iRatio\:\cdot \:{C}_{I,Dual,E}}\end{aligned}$$

These equations enabled direct comparisons of apparent WRs derived from dual-window counts and corrected WRs under clinical dual-isotope acquisition conditions.

### Calculation of counts in clinical study

Myocardial counts from anterior images were measured for planar analysis. A circular region of interest (ROI) was placed over the cardiac region on ^201^Tl images acquired in the 71 keV window. The same ROI was then copied to the corresponding ^123^I-BMIPP image acquired in the 159 keV window to ensure spatial consistency. We calculated WRs for apparent (uncorrected) and spill-in corrected values using the spectral separation model described.

Reconstructed short-axis SPECT images were used to generate polar maps spanning the basal to apical regions of the myocardium. Images were processed using vendor-provided software (GE Healthcare, Tokyo, Japan) using FBP and OSEM reconstruction. The mean myocardial count derived from the polar map served as the representative value for WRs, which were calculated as:

$$\:\:WR\:=\:\:\:\:\:\frac{{C}_{early}-{C}_{late}\cdot \:DCF\:}{{C}_{early}}$$

where *C*_*early*_ and *C*_*late*_ indicate mean polar map counts from the early and late scans, respectively. Relationships between planar myocardial counts and polar map counts, and between planar and SPECT-derived WRs, were evaluated by comparing these parameters.

### Graphic display and statistical analysis

All quantitative data are presented as mean ± standard deviation (SD). Relationships between corrected and uncorrected values and between planar and SPECT-derived WRs were evaluated using correlation coefficients and linear regression analyses. Linear regression lines were displayed to facilitate comparisons of tracer separation performance and WR reproducibility. Statistical significance was defined as *p* < 0.05. Energy spectra, simulated dual-tracer spectral overlap, and scatter plots for WR comparisons were generated using the Wolfram Language version 14.3 (Wolfram Research, Inc., Champaign, IL, USA) and JMP v.18.2 (SAS Institute Inc., Cary, NC, USA).

## Results

### Energy spectra of ^123^I and ^201^Tl in phantom and patient studies

Energy spectra were acquired under single-tracer conditions in phantom experiments and clinical patient studies to characterize spectral overlap between ^123^I and ^201^Tl. Distinct photopeaks for ^123^I (159 keV) and ^201^Tl (≈ 71 keV) were observed in phantom spectra, confirming appropriate energy window settings. Based on the phantom spectra, the spill-in iRatio and tRatio were 0.33 and 0.10, respectively (Fig. 1B).

The overall spectral profiles remained consistent at early and late time points in clinical data derived from three patients (six studies). Shoulder counts slightly increased in patient spectra compared with the phantom (Fig. 1C), which likely reflected scatter and Compton contributions. Nonetheless, peak positions and shapes remained stable among all six studies (Fig. 1D). These findings supported the reproducibility of energy distribution in vivo.

**Fig. 2:**
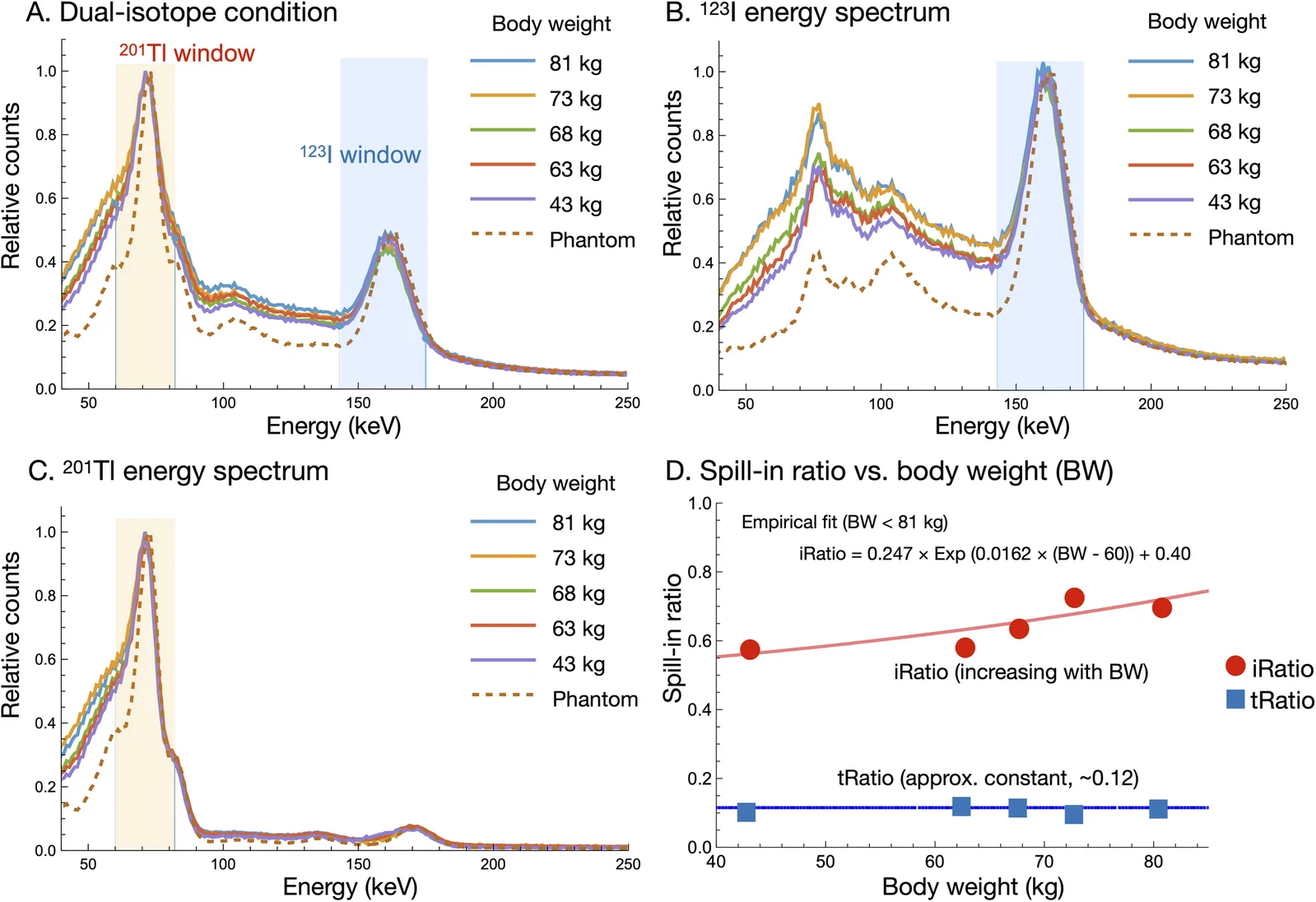
Patient-derived energy spectra and dependence of spill-in ratios on body weight. Energy spectra from five patients weighing 43, 63, 68, 73 and 81 kg acquired under conditions of (**A**) dual-isotope (^123^I + ^201^Tl), (**B**) ^123^I, and (**C**) ^201^Tl. (**D**) Relationship between body weight and spill-in ratios (iRatio and tRatio) derived from energy spectra. Shaded areas in panels A and B indicate acquisition windows. ^123^I-BMIPP, ^123^I-β-methyl-iodophenyl-pentadecanoic acid

Scatter fractions were higher in energy spectra obtained under dual-nuclide conditions for ^123^I and ^201^Tl in patients weighing 43, 63, 68, 73, and 81 kg (Fig. 2) than in phantom spectra. The scatter contribution of ^201^Tl in the intermediate energy range between the ^201^Tl and ^123^I peaks increased with body weight, resulting in a weight-dependent increase in spill-in from ^123^I into the ^201^Tl window. The iRatio range was accordingly 0.57–0.73. In contrast, spill-in from ^201^Tl into the ^123^I window was relatively stable, with a tRatio of 0.10–0.12. Figure 2D shows that tRatio remained approximately constant (~ 0.12), whereas iRatio increased with body weight. The relationship between the iRatio and body weight was approximated using the following empirical model (body weight < 81 kg):

6 $$\begin{aligned}\:iRatio\hspace{0.17em}&=\hspace{0.17em}0.247\:\times\:\:Exp\:[0.0162\:\\&\times\:\:(body\:weight\hspace{0.17em}-\hspace{0.17em}60\left)\right]\hspace{0.17em}+\hspace{0.17em}0.40.\end{aligned}$$

However, as shown in Fig. 2, the fit was not perfect, and the equation should be interpreted as an approximation rather than a strict predictive model. Because the corrected ^123^I-BMIPP washout rate (IWR_estimated_) depended primarily on the tRatio (Eq. 5.1), the observed stability of tRatio supported the use of a fixed spill-in ratio for subsequent dual-tracer corrections. Accordingly, in subsequent calculations, we used a fixed tRatio of 0.12 and a representative iRatio (median 0.64). These empirically determined ratios, derived from clinical data, were applied in all subsequent analyses to separate tracer counts and calculate corrected WRs in planar and SPECT images under dual-isotope conditions.

### Calculation of estimated ^201^Tl and ^123^I counts

True radionuclide-specific counts were estimated using the fixed spill-in ratios and solving the simultaneous equations described in the Methods section. This correction enabled the separation of dual-tracer contributions within each energy window. Table 1 shows the count separation results derived from early and late planar and SPECT images of a representative patient. After applying the spill-in ratios, tracer-specific counts were obtained for both planar and SPECT datasets. The difference between apparent and corrected ^123^I WRs was small in this patient (planar and SPECT images: 18% vs. 17% and 21% vs. 20%, respectively).

### Comparison of corrected and uncorrected washout rates

**Fig. 3:**
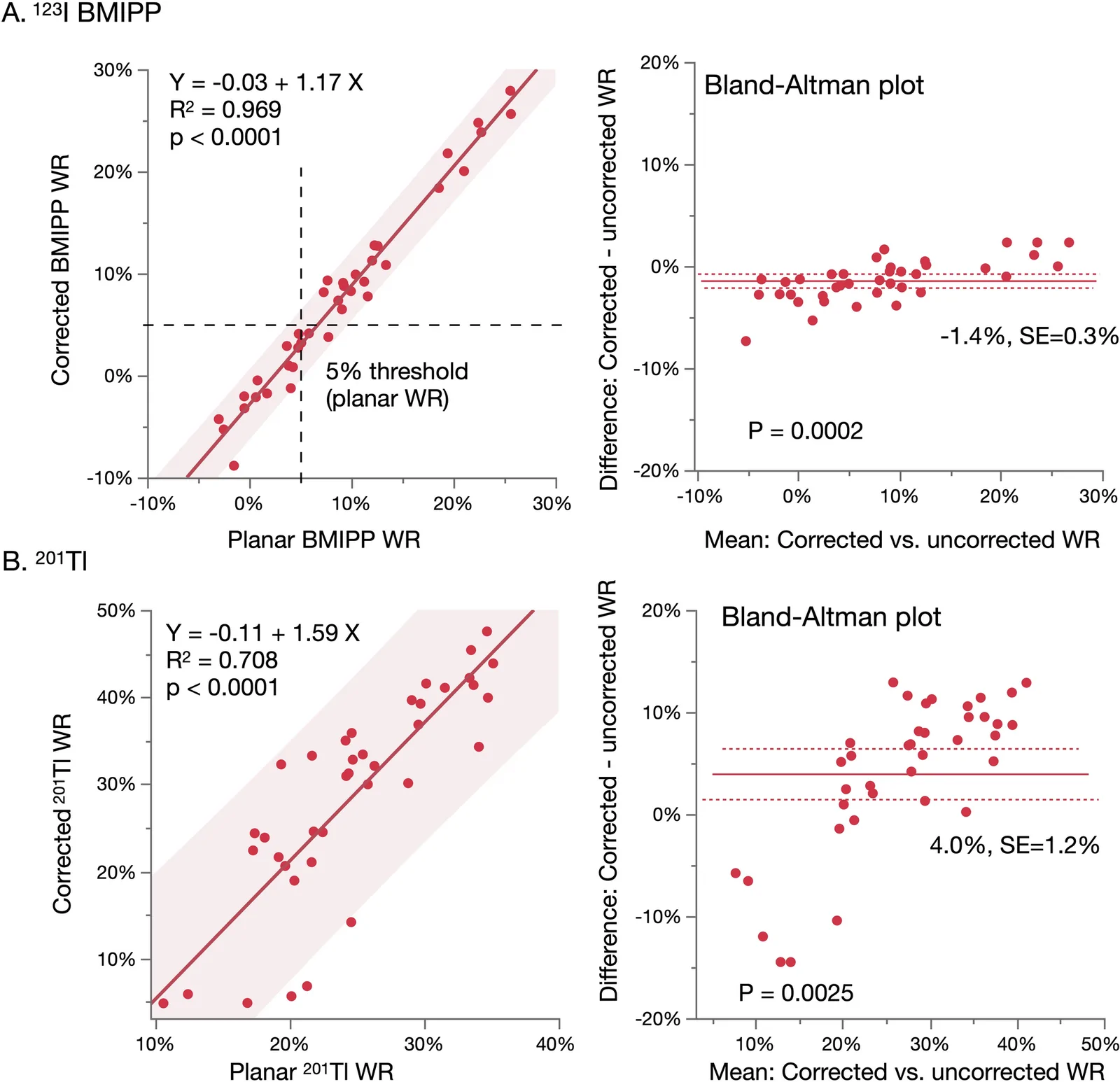
Comparison of uncorrected and corrected planar WRs. Scatter plots show relationships between uncorrected and corrected WRs for (**A**) ^123^I-BMIPP and (**B**) ^201^Tl, with linear regression lines. Right panels show the corresponding Bland–Altman plots. Dashed lines in panel A indicate the 5% WR threshold used for classification. ^123^I-BMIPP, ^123^I-β-methyl-iodophenyl-pentadecanoic acid; WR(s), washout rate(s)

The corrected and uncorrected WRs for ^123^I-BMIPP (*p* < 0.0001) were strongly correlated after applying spectral separation to correct cross-contamination (Fig. 3A). Bland–Altman analysis showed a small negative bias (corrected – uncorrected), with a mean difference of -1.4% ± (SE) 0.3%. In contrast, the ^201^Tl WRs showed only moderate correlation between corrected and uncorrected data, and the corrected WRs of ^201^Tl were unstable (R^2^ = 0.708, *p* < 0.0001; Fig. 3B). Even when a body weight -dependent iRatio was applied (Eq. 6), the correlation remained similar (R^2^ = 0.684, *p* < 0.0001), indicating no substantial improvement with the use of a fixed iRatio (0.64).

**Fig. 4:**
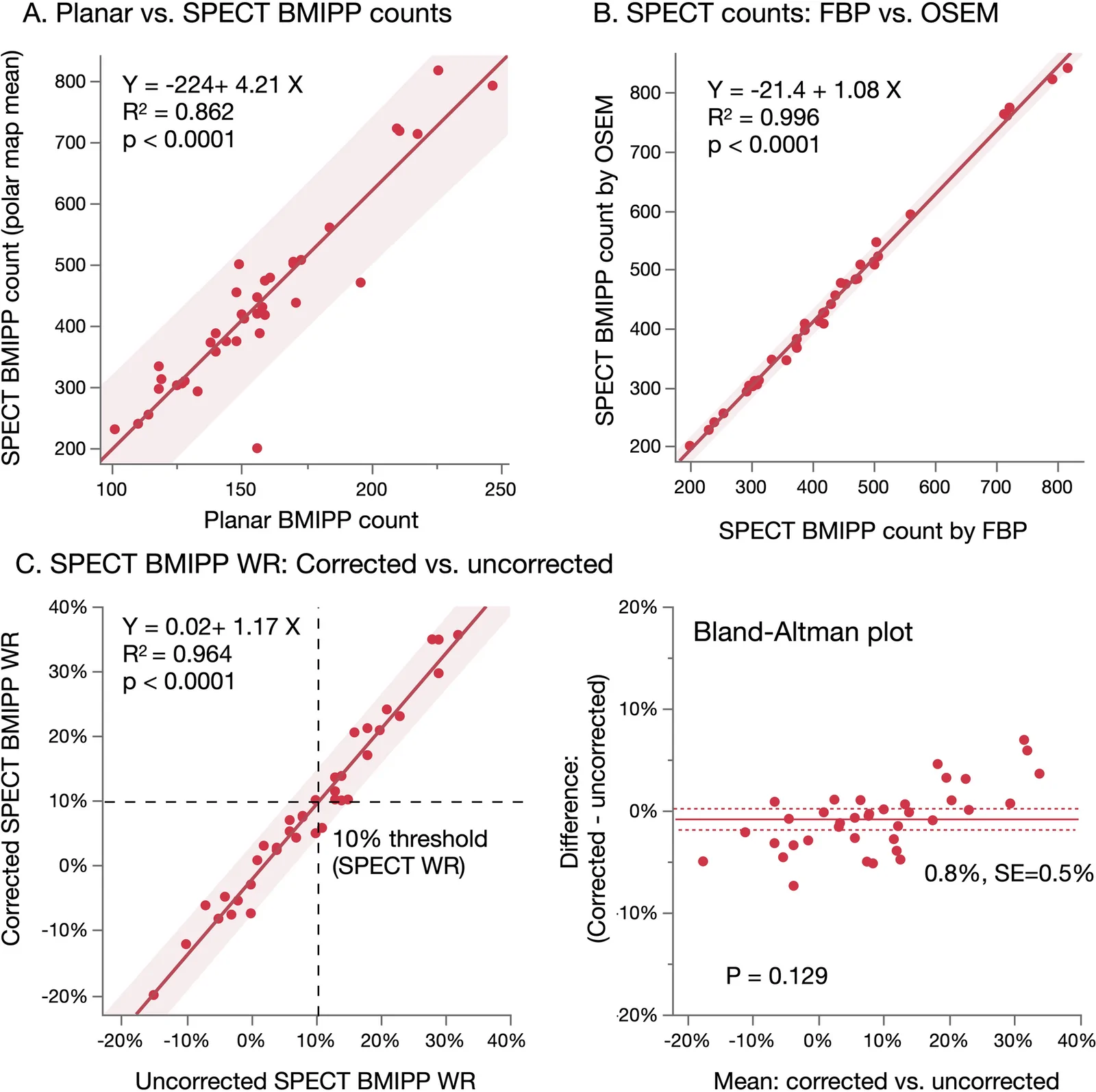
Relationships between planar and SPECT counts and WRs for ^123^I-BMIPP. (**A**) Correlation between anterior planar myocardial counts and mean polar map counts derived from SPECT images. (**B**) Comparison of BMIPP counts from SPECT images reconstructed using FBP and OSEM. (**C**) Right panel shows the relationship between uncorrected and corrected SPECT BMIPP WRs, with the corresponding Bland–Altman plot. Dashed lines in panel C indicate the reference 10% WR threshold. ^123^I-BMIPP, FBP, filtered back projection; OSEM, ordered-subset expectation maximization, WRs, washout rates

Because of this instability, further analyses focused on ^123^I-BMIPP SPECT-derived counts. The comparison of planar and SPECT-derived myocardial counts further supported the validity of the correction approach. Counts derived from anterior planar images and averaged from SPECT polar maps closely correlated; the relationships between SPECT and planar counts were as follows: R^2^ = 0.862; SPECT = -224 + 4.21 × planar count for ^123^I-BMIPP (Fig. 4A), and R^2^= 0.801; SPECT = -157 + 3.12 × planar count for ^201^Tl (*p* < 0.0001 for all).

Correlations between SPECT counts corrected by FBP and OSEM reconstruction were excellent (R^2^ = 0.996, Fig. 4B) and remained high when WR values derived from SPECT were compared before and after correction (R^2^ = 0.964; *p* < 0.0001; Fig. 4C). Bland–Altman plots revealed minimal differences between corrected and uncorrected WRs (0.8%, *p* = 0.129).

### Threshold-based discrepancy analysis

We assessed the potential clinical impact of spectral cross-talk on WR classification using the threshold criteria of 5% (planar) and 10% (SPECT) to classify ^123^I-BMIPP washout as normal or abnormal [10]. Classification agreement was evaluated before and after spill-in correction.

Corrected and uncorrected planar WR values resulted in discordant classification in only 3 (8%) of 38 studies. All discrepancies occurred near the 5% cutoff, indicating that crosstalk correction may influence interpretation primarily in borderline cases. A comparison of corrected planar and SPECT WRs based on their respective thresholds revealed discordant classification in 3 (8%) of 38 studies, again clustered near the threshold values.

## Discussion

We developed a practical reproducible method for correcting spectral cross-talk between ^123^I and ^201^Tl in dual-isotope imaging. Fixed spill-in ratios derived from energy spectra (iRatio for spill-in of ^123^I into the ^201^Tl window and tRatio for spill-in of ^201^Tl into the ^123^I window) allow estimation of tracer-specific counts and corrected WRs that could be calculated without requiring additional hardware or repeated patient-specific energy spectral acquisition. This approach is particularly relevant in clinical settings where accurate ^123^I-BMIPP WR quantitation is required, such as TGCV evaluations and myocardial metabolic impairment assessments after ischemia. The simplicity and robustness of our proposed method support wider clinical implementation of dual-tracer protocols.

### Combination of ^123^I-labeled radiopharmaceuticals and ^201^Tl

Dual-isotope imaging with ^123^I-labeled radiopharmaceuticals and ^201^Tl has been widely applied in Japan to evaluate ischemic heart disease, as ^123^I-BMIPP determines myocardial fatty acid metabolism and ^201^Tl defines cardiac perfusion. The high sensitivity of ^123^I-BMIPP allows the detection of metabolic abnormalities in acute coronary syndrome and spasm-related ischemia [13, 14]. During the acute phase particularly after successful coronary intervention, mismatch patterns characterized by preserved ^201^Tl uptake and reduced ^123^I-BMIPP uptake indicate viable, but stunned myocardium, and may predict subsequent functional recovery. Similarly, BMIPP defects in vasospastic angina often correspond to vasospastic territories and normalize together with recovered left ventricular contractility after appropriate therapy [15, 16].

The Japanese Circulation Society (JCS) guidelines support the application of ^123^I-BMIPP SPECT with Class I or IIa indications for diagnosing myocardial ischemia, evaluating vasospastic angina, and assessing myocardial damage in patients undergoing hemodialysis [17]. Although separate acquisitions for metabolism and perfusion are feasible, simultaneous dual-isotope imaging with ^123^I-BMIPP and ^201^Tl remains clinically attractive because it enables efficient assessment of mismatch patterns in time-sensitive settings.

Subtraction-based approaches and multi-window correction methods have been applied to isolate tracer activity to address photon energy overlap between ^123^I and ^201^Tl. While these methods support visual interpretation, their impact on quantitative indices such as WRs has not been systematically validated. We addressed this gap by quantifying the influence of spectral crosstalk and correction on WR measurements in phantom and clinical datasets.

Dual-isotope imaging has also been applied to combinations of ^123^I-*meta*-iodobenzylguanidine (MIBG) and ^201^Tl, enabling assessment of perfusion–innervation mismatch. This mismatch has clinical value for risk stratification in heart failure, particularly for predicting sudden cardiac death and ventricular arrhythmias [18, 19]. Therefore, the proposed correction framework may be applicable to other ^123^I/^201^Tl dual-isotope protocols such as ^123^I-MIBG studies, although radionuclide- and system-specific determination of spill-in ratios would be required.

### Late ^123^I-BMIPP images and washout rates

Late-phase ^123^I-BMIPP images are typically acquired 3 h after a radiolabeled tracer is injected into a patient. This approach was initially explored during early clinical investigations in the 1990s but was not widely adopted into routine practice. This was largely due to the predominant clinical emphasis on detecting mismatch patterns between fatty acid metabolism (^123^I-BMIPP) and perfusion [1, 20, 21], rather than focusing on quantitative indices such as WRs. Consequently, clinical use of late BMIPP imaging gradually declined in most institutions.

However, interest in WR quantitation has been revitalized with the recognition of TGCV as a distinct clinical entity [9, 22–24]. A viable myocardium rapidly takes up and incorporates ^123^I-BMIPP into the intracellular triglyceride pool, where it is subsequently cleared over time. Therefore, WRs provide a quantitative measure of the dynamic status of myocardial lipid metabolism.

### Proposed correction method using energy-spectrum analysis

Our proposed approach assumes that spill-in ratios between radionuclides remain sufficiently stable under clinical imaging conditions to allow mathematical decomposition of measured counts using fixed coefficients. This assumption is supported by the finding that overall energy-spectral patterns are reproducible *in vivo.* In this study, the tRatio (spill-in of ^201^Tl into the ^123^I window) remained relatively stable among patients, whereas iRatio (spill-in of ^123^I into the ^201^Tl window) varied with body habitus. This variability is likely attributable to differences in scatter conditions, which are influenced not only by body weight but also by stature and attenuation geometry, as shown by ^201^Tl spectral variation (Fig. 2B). Although alternative contamination indices such as C_T, wI_/C_I, wI_ or C_I, wT_/C_T, wT_ ratios could also be defined, they were not adopted because they depend on relative administered activities and myocardial ^201^Tl and ^123^I-BMIPP uptake, which may substantially vary among patients. In contrast, the spill-in ratios derived from energy spectra in our model were less sensitive to inter-patient variability in tracer uptake.

Spill-in ratios were derived from clinical energy spectra rather than phantom measurements. The apparent ^201^Tl peak detected around 71 keV is a composite, rather than a single discrete photon energy, reflecting characteristic mercury X-rays (Kα = 70.8 keV, 73.7%; Kβ = 80.3 keV, 20.4%) as well as additional characteristic X-rays generated by interactions with the lead collimator (Pb Kα ≈ 75 keV; Pb Kβ ≈ 85 keV). Therefore, peak positions and spectral shapes may differ slightly between phantom and patient conditions. However, because spill-in ratios were consistently derived from clinical data, these variations are considered unlikely to significantly affect correction performance.

Optimal iRatios and tRatios might differ among imaging systems and protocols, including detector type (Anger vs. cadmium-zinc-telluride [CZT]), collimator design, energy window settings, and reconstruction parameters. Although agreement between FBP and OSEM reconstructions was excellent, newer CZT cameras with improved energy resolution might further reduce spectral overlap compared with conventional Anger systems, and may improve quantitative accuracy in dual-isotope imaging, including protocol combinations of ^123^I-BMIPP with perfusion tracers [25, 26].

The purpose of this study was not to define universal spill-in ratios, but rather to provide a systematic and clinically practical correction framework. The method can be locally optimized at any institution using simple phantom measurements and a few representative patient spectra. This spill-in ratio–based approach allows dual-isotope protocols to retain both qualitative and quantitative utility, including more accurate WR calculation, even in the presence of overlapping radionuclide energy spectra.

### Corrected versus uncorrected washout rates

This study showed that ^123^I-BMIPP WRs corrected for spectral crosstalk in dual-isotope imaging strongly and consistently correlated with uncorrected values. Corrected WRs derived from planar images were slightly lower on average than apparent WRs, while maintaining a high correlation, supporting the internal consistency of the correction approach. A similarly strong correlation was observed for SPECT-derived WRs. Thus, uncorrected WRs may provide reasonable estimates in many clinical situations. However, in cases near diagnostic thresholds (e.g., TGCV vs. normal), even small differences may influence classification. Therefore, the correction method is valuable for improving diagnostic accuracy and reducing the risk of misclassification in borderline cases. In contrast, ^201^Tl WRs were not sufficiently stable when using simple fixed spill-in ratios and were therefore not further evaluated for quantitative WR analyses. Moreover, variability in iRatio could not be sufficiently compensated by simple body weight–based adjustment.

In clinical settings such as acute coronary syndrome, angina pectoris, and myocardial infarction, WRs near 0% might suggest significantly impaired myocardial fatty acid clearance and possible TGCV. By reducing bias due to spectral overlap, our proposed correction method improves confidence in WR interpretation and enhances the reproducibility of quantitative assessment using dual-isotope ^123^I-BMIPP/^201^Tl protocols.

### Washout rates in TGCV

Defective intracellular lipolysis in TGCV results in low ^123^I-BMIPP clearance (nearly zero), indicating severely impaired fatty acid utilization and metabolic trapping [11, 23]. A ^123^I-BMIPP WR of < 10% has been proposed as part of the diagnostic criteria for TGCV when supported by other characteristic findings such as myocardial triglyceride accumulation on computed tomography or magnetic resonance images, along with angiographic evidence of diffuse concentric coronary narrowing [9, 24, 27]. These elements form the basis of the TGCV diagnostic algorithm established through Japanese clinical research.

The Japanese Society of Nuclear Cardiology (JSNC) and the Japanese Society of Nuclear Medicine (JSNM) have recommended single-isotope acquisition for ^123^I-BMIPP WR analysis to support accurate WR measurements [11]. Although dual-isotope imaging is clinically attractive for simultaneous metabolism and perfusion assessment, it has not been recommended for WR quantitation because spectral cross-talk between ^123^I and ^201^Tl can introduce substantial bias in tracer separation and WR calculations.

Nevertheless, some institutions have applied dual-isotope ^123^I-BMIPP and ^201^Tl protocols during the early phase of TGCV research, before correction methods were standardized. The present findings provide a reproducible mathematical framework for separating ^123^I and ^201^Tl counts using empirically derived energy spectra, which allows estimation of ^123^I-BMIPP WRs under dual-isotope conditions. This approach may improve the interpretability of archived dual-isotope datasets and also support quantitative analysis in settings where dual-isotope acquisition is used for clinical efficiency.

To assess potential clinical impact, we applied previously proposed cutoff values for abnormal WR (5% for planar imaging and 10% for SPECT) [10]. Classification discrepancies between corrected and uncorrected WR values were uncommon when the 10% threshold was used. However, misclassification may occur primarily near decision thresholds, indicating that correction may influence interpretation mainly in borderline cases. Moreover, when dual-nuclide studies are performed in patients with coronary artery disease or heart failure, simultaneous calculation of corrected WRs may obviate the need for an additional ^123^I-BMIPP study. The proposed method also provides a practical means to re-evaluate previously acquired datasets, thereby preserving their clinical and research utility, particularly in the context of TGCV.

Finally, although several studies in this cohort had ^123^I-BMIPP WRs < 10%, TGCV cannot be inferred from WR alone. Diagnostic confirmation requires multimodal imaging and clinical correlation. Because clinical experience with ^123^I-BMIPP WR remains limited, further evaluation across diverse clinical and pathophysiological conditions is warranted.

### Limitations and future directions

Several limitations should be acknowledged. This study, conducted at a single-center, obtained all energy spectra, spill-in ratios, and validation analyses using a single gamma camera system with fixed acquisition and reconstruction protocols. Therefore, generalizability to other institutions, camera models, and imaging conditions remains to be established.

Our proposed correction for ^123^I-BMIPP WR relies primarily on tRatio in both planar and SPECT imaging. Because SPECT reconstruction introduces depth-dependent attenuation and scatter effects, spill-in might vary spatially within the myocardium. Although agreement between FBP and OSEM reconstructions was excellent, other reconstruction approaches (e.g., resolution recovery, attenuation correction, or more sophisticated scatter correction models) might differently influence spill-in.

In addition, ^201^Tl WR could not be reliably quantified using fixed iRatio values, and iRatio variability related to body habitus was not evaluated in patients weighing > 81 kg. Although iRatio was associated with body weight, scatter within the body is influenced by multiple factors beyond body weight alone, which may contribute to its instability. This may explain why body weight–based adjustment did not sufficiently improve the reliability of ^201^Tl WR. In contrast, tRatio (spill-in from ^201^Tl into the ^123^I window) was relatively stable and can be used for calculating ^123^I-BMIPP WRs.

For a more rigorous comparison, data from both single-isotope and dual-isotope studies would be desirable. However, in clinical practice, it was not feasible to perform repeated acquisitions using both protocols in the same patients because of ethical and logistical constraints, including additional radiation exposure.

Although patient spectra were generally consistent in our cohort, spectral characteristics may differ among institutions depending on detector type (Anger vs. CZT), collimator design, energy resolution, and energy window settings. These factors might affect the applicability of fixed spill-in ratios when implemented under various clinical conditions.

Finally, while the present study demonstrates the feasibility of a practical correction framework, further investigation is required to validate and optimize the approach across diverse imaging systems and patient populations. Multicenter studies are needed to establish system-specific correction factors and to develop practical recommendations for routine clinical implementation of dual-isotope protocols.

## Conclusion

This study demonstrated that correcting spectral crosstalk between ^123^I and ^201^Tl in dual-isotope imaging using fixed, energy spectrum–derived spill-in ratios is feasible and robust. The proposed method enables practical WR quantitation under dual-tracer acquisition conditions without requiring patient-specific spectral measurements. This approach supports reliable quantitative assessment, particularly in threshold-based interpretation, and may facilitate broader clinical use of dual-isotope protocols for myocardial ischemia and TGCV.

**Table 1:** Examples of ^123^I and ^201^Tl counts separation from dual-window measurements

	^201^Tl (71 keV, 30%)	^123^I (159 keV, 20%)
*Spill-in ratios*		
iRatio of ^123^I to ^201^Tl window	0.64	-
tRatio of ^201^Tl to ^123^I window	-	0.12
*Planar study: anterior images*		
Early/late counts	265/183	218/153
Early/late corrected counts	136/92	202/142
Apparent WR (uncorrected, %)	29%	18%
Corrected WR (spill-in corrected, %)	30%	17%
*SPECT study: polar maps*		
Early/late counts	794/497	713/480
Early/late corrected counts	366/206	669/455
Apparent WR (uncorrected, %)	36%	21%
Corrected WR (spill-in corrected, %)	42%	20%

WR, washout rate

## Electronic Supplementary Material

Below is the link to the electronic supplementary material.

Supplementary Material 1

## Data Availability

The datasets generated during and/or analyzed during the current study are available from the corresponding author on reasonable request.

## Abbreviations

BMIPP: β-methyl-iodophenyl pentadecanoic acid
CZT: Cadmium-zinc-telluride
FBP: Filtered back projection
OSEM: Ordered-subset expectation maximization
SPECT: Single-photon emission computed tomography
TGCV: Triglyceride deposit cardiomyovasculopathy
WR: Washout rate
