# Supplementary material for: Energy spectrum-based correction of washout rates in dual-isotope 123I-BMIPP/201Tl imaging
Source: Ann Nucl Med. 2026 May 30;40(10):1197–209. doi: 10.1007/s12149-026-02235-8 (PMC13619768; doi:10.1007/s12149-026-02235-8)
Supplement: Supplementary file 1 [file 12149_2026_2235_MOESM1_ESM.pdf]

Supplementary Derivation of Spill-in Correction and Washout Rate Equations

Step-by-step mathematical derivation for clarity

Energy Spectrum-Based Correction of Washout Rates in Dual-Isotope ^{123}I-BMIPP/^{201}Tl imaging

Kenichi Nakajima, Kanazawa University

1. Definitions

C_I = true ^{123}I counts in the ^{123}I window

C_T = true ^{201}Tl counts in the ^{201}Tl window

$C_{I,Dual}$ = measured counts in the ^{123}I window under dual-isotope acquisition

$C_{T,Dual}$ = measured counts in the ^{201}Tl window under dual-isotope acquisition

$iRatio = C_{I,wT} / C_{I,wI}$

$tRatio = C_{T,wI} / C_{T,wT}$

2. Measurement model

$$C_{I,Dual} = C_I + C_T \cdot tRatio \qquad (S1)$$

$$C_{T,Dual} = C_T + C_I \cdot iRatio \qquad (S2)$$

3. Derivation of separated true counts

From Eq. (S1),

$$C_I = C_{I,Dual} - C_T \cdot tRatio \qquad (S3)$$

Substitute Eq. (S3) into Eq. (S2):

$$C_{T,Dual} = C_T + (C_{I,Dual} - C_T \cdot tRatio) \cdot iRatio$$

$$= C_T + C_{I,Dual} \cdot iRatio - C_T \cdot iRatio \cdot tRatio$$

$$= C_T \cdot (1 - iRatio \cdot tRatio) + C_{I,Dual} \cdot iRatio$$

Therefore,

$$C_T = (C_{T,Dual} - C_{I,Dual} \cdot iRatio) / (1 - iRatio \cdot tRatio) \qquad (S4)$$

By symmetry,

$$C_I = (C_{I,Dual} - C_{T,Dual} \cdot tRatio) / (1 - iRatio \cdot tRatio) \qquad (S5)$$

4. Definition of washout rate

$$WR = (C_E - C_L \cdot DCF) / C_E \qquad (S6)$$

$$DCF = 1 / 0.5^\wedge(\Delta t / HL) \qquad (S7)$$

where CE and CL denote early and late counts, Δt is the interval between scans, and HL is the physical half-life.

5. Apparent washout rates under dual-isotope acquisition

$$IWRapp = (C_{I,Dual,E} - C_{I,Dual,L} \cdot DCFI) / C_{I,Dual,E} \qquad (S8)$$

$$TWRapp = (C_{T,Dual,E} - C_{T,Dual,L} \cdot DCFT) / C_{T,Dual,E} \qquad (S9)$$

6. Corrected washout rate for ^{123}I-BMIPP

$$C_{I,E} = (C_{I,Dual,E} - tRatio \cdot C_{T,Dual,E}) / (1 - iRatio \cdot tRatio) \qquad (S10)$$

$$C_{I,L} = (C_{I,Dual,L} - tRatio \cdot C_{T,Dual,L}) / (1 - iRatio \cdot tRatio) \qquad (S11)$$

Insert Eqs. (S10) and (S11) into Eq. (S6):

$$IWR_{estimated} = (C_{I,E} - C_{I,L} \cdot DCF_I) / C_{I,E} \qquad (S12)$$

After cancellation of the common denominator,

$$IWR_{estimated} = [(C_{I,Dual,E} - C_{I,Dual,L} \cdot DCF_I) - tRatio \cdot (C_{T,Dual,E} - C_{T,Dual,L} \cdot DCF_I)] / (C_{I,Dual,E} - tRatio \cdot C_{T,Dual,E}) \qquad (S13)$$

7. Corrected washout rate for ^{201}Tl

$$C_{T,E} = (C_{T,Dual,E} - iRatio \cdot C_{I,Dual,E}) / (1 - iRatio \cdot tRatio) \qquad (S14)$$

$$C_{T,L} = (C_{T,Dual,L} - iRatio \cdot C_{I,Dual,L}) / (1 - iRatio \cdot tRatio) \qquad (S15)$$

Insert Eqs. (S14) and (S15) into Eq. (S6):

$$TWR_{estimated} = (C_{T,E} - C_{T,L} \cdot DCF_T) / C_{T,E} \qquad (S16)$$

After cancellation of the common denominator,

$$TWR_{estimated} = [(C_{T,Dual,E} - C_{T,Dual,L} \cdot DCFT) - iRatio \cdot (C_{I,Dual,E} - C_{I,Dual,L} \cdot DCFT)] / (C_{T,Dual,E} - iRatio \cdot C_{I,Dual,E}) \qquad (S17)$$

8. Practical implication

Eq. (S13) shows that the corrected ^{123}I washout rate is determined primarily by tRatio, whereas Eq. (S17) indicates that the corrected ^{201}Tl washout rate depends on iRatio, which sgiws greater variability across patients.
